# Real-world outcome of neoadjuvant therapy with or without pembrolizumab for triple-negative breast cancer

**DOI:** 10.2340/1651-226X.2026.44896

**Published:** 2026-02-19

**Authors:** Meeri Maunu, Juho Lähteenmaa, Peeter Karihtala, Suvi Tuohinen, Joonas Laaksolilja, Anders Ståhls, Tuomo Meretoja, Johanna Mattson

**Affiliations:** a University of Helsinki, Doctoral Education Pilot in Precision Cancer Medicine, Helsinki, Finland; b Department of Helsinki University Hospital IT Management, AI and Data Analytics, Helsinki, Finland; c Comprehensive Cancer Center, Helsinki University Hospital and University of Helsinki, Helsinki, Finland; d Helsinki University Hospital, Heart and Lung Center, and University of Helsinki, Finland; e Helsinki University Hospital, Diagnostic Center, Department of Pathology, Helsinki, Finland; f Division of Breast Surgery, Comprehensive Cancer Center, Helsinki University Hospital, and University of Helsinki, Helsinki, Finland

**Keywords:** triple-negative breast cancer, neoadjuvant therapy, pembrolizumab, real-world evidence, effectiveness, adverse effects

## Abstract

**Background and purpose:**

Neoadjuvant therapy (NAT) has become standard therapy for early triple-negative breast cancer (TNBC). The aim of this study was to report real-world outcome of TNBC treated with NAT with or without pembrolizumab and to identify predictive factors for achieving pathologic complete response (pCR) in the pembrolizumab cohort.

**Patient/material and methods:**

The data of 75 consecutive TNBC patients treated with neoadjuvant chemotherapy and pembrolizumab at the Helsinki University Hospital Comprehensive Cancer Center were retrospectively collected. Treatment outcome and predictive factors for pCR were analyzed. Additionally, the outcome of nonmatched 102 consecutive TNBC patients treated without pembrolizumab during the preceding years is reported.

**Results:**

Forty-two patients (56.0%) achieved pCR to pembrolizumab-based NAT, while 47 patients (46.1%) without pembrolizumab had pCR. Lymph node metastasis (*p* = 0.011) and multifocality (*p* < 0.001) were inversely associated with pCR in the pembrolizumab cohort. Thirty-four patients (45.3%) had immune-related adverse events (irAEs), and 11 patients (14.7%) had grade 1–2 myocarditis in the pembrolizumab cohort. Due to adverse events (AEs), pembrolizumab was discontinued in 22 patients (29.3%) in the neoadjuvant setting, not started postoperatively in 21 patients (28%) and discontinued postoperatively in eight patients (10.7%). The number of preoperative pembrolizumab cycles was not associated with pCR.

**Interpretation:**

Despite higher incidence of myocarditis and interruption of the systemic therapy due to irAEs, higher pCR rates were seen with pembrolizumab. Even though the number of preoperative pembrolizumab cycles was not associated with pCR monitoring and limiting AEs is important.

## Introduction

Triple-negative breast cancer (TNBC) represents an aggressive subtype of breast cancer [1]. It accounts for approximately 10–20% of all breast cancer cases. Characterized by the absence of expression of estrogen receptor (ER), progesterone receptor (PR), and human epidermal growth factor receptor 2 (HER2), TNBC is more challenging to treat, as these receptors also serve as targets for systemic therapy. TNBC has limited treatment options and is associated with a poorer prognosis compared to other breast cancer subtypes [2].

Neoadjuvant therapy (NAT) has become the standard of care for early-stage TNBC, offering the benefits of tumor downstaging, surgical de-escalation, and possibilities to tailor adjuvant treatment according to treatment response [3, 4]. Achieving pathologic complete response (pCR) after NAT is associated with improved long-term outcomes, including disease-free survival (DFS) and overall survival (OS) in TNBC [5, 6]. However, the high heterogeneity and chemoresistance of TNBC limit the effectiveness of NAT in a significant subset of patients [7]. In a large retrospective registry-based study from Sweden, comparable survival was seen among patients with TNBC treated with neoadjuvant or adjuvant chemotherapy [8].

Recent advances in immunotherapy, especially immune checkpoint inhibitors (ICI), have transformed the therapeutic landscape for TNBC [9]. Pembrolizumab, a monoclonal antibody targeting the programmed cell death protein 1 (PD-1) receptor, has emerged as a pivotal agent in the neoadjuvant and adjuvant settings for TNBC. Clinical trials have demonstrated that the addition of pembrolizumab to standard NAT regimens significantly increased pCR rates, event-free survival (EFS), and OS in patients with early-stage TNBC [10–12]. Despite its promising efficacy, the integration of pembrolizumab into NAT regimens poses challenges, including the management of immune-related adverse events (irAEs) [10, 13, 14].

This study aimed to report the real-world (RW) clinical outcome of consecutive early TNBC patients treated with pembrolizumab containing neoadjuvant systemic therapy at the Helsinki University Hospital (HUS) Comprehensive Cancer Center (CCC), Finland. EMA approved pembrolizumab an indication for neoadjuvant and adjuvant treatment of TNBC in May 2022. At our hospital, pembrolizumab was taken in use for this indication in December 2022. Secondary aim was to identify predictive factors for achieving pCR. In addition, the outcome of NAT without pembrolizumab for consecutive patients with early TNBC during the preceding years or due to contraindication for pembrolizumab was analyzed.

## Patients/material and methods

This study was reported according to the STROBE guidelines [15].

### Study design

This study is a retrospective single center study on the real-world evidence of NAT in patients with early-stage TNBC treated at the HUS CCC, Finland. For effectiveness outcomes, data of patients with early TNBC treated with NAT without pembrolizumab were collected during the preceding or same years if there was a contraindication for pembrolizumab.

### Patients

Consecutive patients who underwent NAT, including pembrolizumab and surgery before September 1, 2024, were included in the pembrolizumab cohort. The cohort of patients treated with NAT without pembrolizumab included nonmatched consecutive patients with early-stage TNBC diagnosed since January 1st, 2021 and operated on by September 1st, 2024. Most of these patients were treated before pembrolizumab was taken in use for early-stage TNBC at the HUS.

In addition to breast imaging, a computed tomography scan of the body was taken before NAT to rule out distant metastases.

All the patients had breast cancer surgery at the HUS.

### Data collection

The data were collected through a combination of automated data extraction and manual collection. Eligible cases were identified through codes as described in the EU-funded Oncovalue project GitHub repository [16]. Automatically extracted data were subsequently validated by the clinical team. The data that were not possible to extract automatically were collected manually.

**Figure 1:**
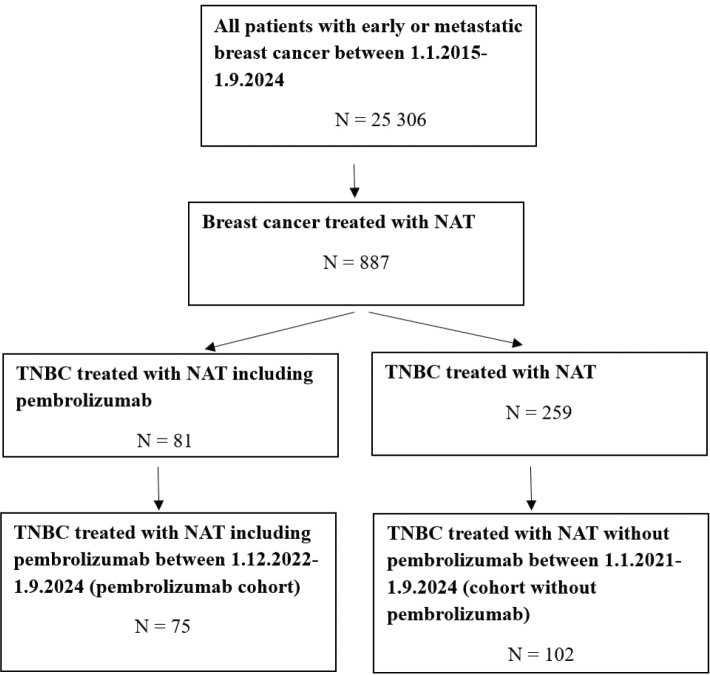
Flowchart diagram of case selection process of analysis. NAT: neoadjuvant therapy; TNBC: triple-negative breast cancer.

Clinical data were collected in both cohorts until May 21st, 2025. Both cohorts also included patients with ER-low (1–10%) and HER2-low (immunohistochemistry [IHC] 1+ or 2+ and in situ hybridization [ISH] negative) tumors. The patient selection process is described in Figure 1. Six patients were excluded from the pembrolizumab cohort. Of these excluded, two patients had early local recurrence after previous adjuvant systemic therapy for TNBC. One patient had already undergone breast surgery, one patient had ER-positive BC (ER 30%) with local recurrence and unknown ER status while one patient had ER-positive BC (ER 15%). One patient had HER2-positive BC and was treated accordingly in the beginning of NAT. All 75 patients were treated in accordance with clinical standards.

### Characterization of tumors and assessment of response

The pathological characteristics of the tumors were determined prior to the initiation of NAT on core needle samples. All the samples were examined in the department of pathology at the HUS. ER and PR negativity were defined as <1% of the cells expressing hormonal receptors via an IHC analysis. ER-low was defined as 1–10% ER-positivity by IHC. HER2 status was prescreened by IHC and considered negative if IHC was 0 and as HER2-low if IHC was 1+. IHC 2+ and 3+ cases were verified by in situ hybridization, and the HER2-status was considered low, when nonamplified in ISH. The clinical tumor size was defined as the largest diameter on MRI, if available. In multifocal tumors, the size was defined by the diameter of the largest focus. MIB-1 score was defined as the percentage of dividing tumor cells.

Response to NAT was evaluated by breast pathologists using surgical breast samples. pCR was defined according to the American Joint Committee on Cancer (AJCC) as no invasive cancer left in both the primary tumor and lymph nodes after NAT (ypT0/is ypN0). Postoperative histopathological characteristics were assessed from the residual tumor samples or lymph nodes. Radiologic complete response (rCR) was defined by breast radiologists, and rCR was defined as the disappearance of the tumor on imaging before surgery.

### Adverse events

Adverse events (AEs) were collected in the pembrolizumab cohort throughout the NAT and for 30 days after discontinuation of pembrolizumab. Treatment-related toxicities were reported retrospectively according to the Common Terminology Criteria for Adverse Events v5.0 [17]. AEs and laboratory values were collected retrospectively from medical reports and were graded by the clinical researchers. Myocarditis was defined according to the ESC (European Society of Cardiology) guidelines by a cardiologist [18]. Electrocardiogram (ECG) was taken before the start of NAT and Troponin before every pembrolizumab cycle. In case of abnormal test or cardiac symptoms, cardiac imaging was done. According to ESC guidelines, ICI myocarditis was diagnosed in case of new troponin elevation with either one major or two minor criteria.

### Statistical analysis

Statistical analyses were conducted using SPSS version 29.0.0.0 (241) (IBM Corporation) in the HUS’ secure analytical environment Acamedic of the HUS. Continuous data are presented as median values with ranges and dichotomous variables as numbers with percentages. Fisher’s exact test was conducted to test the associations of the outcome with dichotomous baseline variables, Fisher-Freeman-Halton exact test for categorical variables with more than two categories, while Mann–Whitney U test was used to test the associations between continuous variables and the outcome. Multivariable logistic regression was used to assess the effect of multiple variables on the achievement of pCR. *P*-values < 0.05 were considered statistically significant. Survival according to treatment response i.e. pCR or on-pCR was analyzed using the Kaplan–Meier method, and the log-rank test was used for comparisons. EFS was calculated from the date of preoperative core needle biopsy to the time of disease progression or the first local or distant recurrence, death or end of the follow-up. Data from patients who did not have an event at the time of data analysis were censored for EFS at the date on which they were last known to be alive and event free.

## Results

A total of 75 patients with early TNBC were treated with pembrolizumab in combination with neoadjuvant chemotherapy between December 2022 and August 2024, and 102 patients with early TNBC were treated with NAT without pembrolizumab between January 2021 and August 2024 at the HUS CCC, Finland.

Baseline characteristics of the study groups are presented in Table 1.

**Table 1:** Baseline characteristics of the study groups.

	Neoadjuvant therapy with pembrolizumab *n* = 75	Neoadjuvant therapy without pembrolizumab *n* = 102
	Median (range)	Median (range)
Age (years)	49 (28–77)	52 (25–78)
	Number (%)	Number (%)
Menopausal status		
Premenopausal	39 (52.0)	49 (48.9)
Postmenopausal	33 (44.0)	47 (46.1)
Unknown	3 (4.0)	6 (5.9)
ECOG performance status score		
0	57 (76.0)	50 (49.0)
1	4 (5.3)	12 (11.8)
Unknown	14 (18.7)	40 (39.2)
gBRCA1/2 predisposition		
Negative	64 (85.3)	91 (89.2)
BRCA1	9 (12.0)	8 (7.8)
BRCA2	2 (2.7)	3 (2.9)
Histology		
Invasive ductal carcinoma	71 (92.2)	91 (89.2)
Invasive metaplastic carcinoma	4 (5.2)	3 (2.9)
Invasive apocrine carcinoma	1 (1.3)	3 (2.9)
Invasive lobular and ductal carcinoma	1 (1.3)	1 (1.0)
Invasive lobular carcinoma	0	2 (2.0)
Invasive medullary carcinoma	0	1 (1.0)
Invasive papillary carcinoma	0	1 (1.0)
Grade		
2	12 (15.6)	11 (10.8)
3	65 (84.4)	74 (72.5)
Unknown	0	17 (16.7)
MIB1 score (%)		
	80 (20–95)	70 (10–95)
Primary tumor diameter mm		
	26 (7–110)	35 (6–110)
Multifocal tumors		
	21 (27.3)	24 (23.5)
Nodal involvement		
Negative	43 (55.8)	43 (42.2)
Positive	34 (44.2)	59 (57.8)
Primary stage		
Stage I	7 (9.1)	5 (4.9)
Stage II	57 (74.0)	60 (58.8)
Stage III	13 (16.9)	24 (23.5)
Unknown	0	13 (12.7)
Estrogen receptor staining		
0*	63 (81.8)	86 (84.3)
1–10%	14 (18.2)	16 (15.7)
HER2 score		
Negative	39 (50.6)	76 (74.5)
1+	21 (27.3)	6 (5.9)
2+	17 (22.1)	20 (19.6)

ECOG: Eastern Cooperative Oncology Group; gBRCA1/2: germline breast cancer gene 1/2 mutation; HER2: human epidermal growth factor receptor 2; ER: estrogen receptor; MIB-1: proliferation marker.

* Less than five patients had bifocal tumors, where one focus was ER 0%, and the other focus was ER greater than 50%. In these cases, the triple negative focus was chosen.

### Characteristics of the patients and tumors in the pembrolizumab cohort

The median age of the patients was 49 years (range 28–77). Thirty-nine patients (52.0%) were premenopausal at diagnosis. Two patients had bilateral BC. A total of 71 tumors (92.2%) were invasive ductal carcinomas, and 65 (84.4%) were classified as grade 3. A total of 47 tumors (61.0%) were classified as T2, and 21 tumors (27.3%) were classified as either bifocal or multifocal. Regarding lymph node involvement, 43 tumors (55.8%) showed no lymph node metastasis in primary diagnostics, including axillary ultrasound and needle biopsies when suspicious nodes were seen. Nodal involvement was confirmed by pretreatment needle biopsies in 34 patients, and additionally two patients were clinically node negative, but they had tumor-positive sentinel lymph nodes at surgery after NAT. Baseline characteristics of the study groups are shown in Table 1. The median MIB1 proliferation score was 80% (range 20–95). Nine patients (12.0%) had breast cancer susceptibility gene BRCA1 (breast cancer gene 1) mutation, and two (2.7%) had BRCA2 (breast cancer gene 2) mutation.

### Oncological and surgical treatments in the pembrolizumab cohort

Table 2 shows the oncological and surgical treatments administered. All patients received neoadjuvant pembrolizumab in combination with a taxane and carboplatin, followed by pembrolizumab combined with AC/EC. Gemcitabine was added in the NAT for one patient due to radiological progression.

**Table 2:** Oncological and surgical treatments of the patients treated with neoadjuvant pembrolizumab.

Number of preoperative pembrolizumab cycles of 75 patients	No. of cycles	No. of patients (%)
	1	3 (4.0)
	2	7 (9.3)
	3	5 (6.7)
	4	6 (8.0)
	5	20 (26.7)
	6	31 (41.3)
	7	1 (1.3)
	8	2 (2.7)
Median number of preoperative pembrolizumab cycles (range)	5 (1–8)	
Surgery for 77 breasts	Type of surgery	
	Breast conserving surgery (BCS) + sentinel lymph node biopsy (SLNB)	28 (36.4)
	BCS + axillary lymph node dissection (ALND)	10 (13.0)
	BCS + targeted axillary dissection (TAD)	9 (11.7)
	BCS + TAD + ALND	1 (1.3)
	Mastectomy + ALND	12 (15.6)
	Mastectomy + SLNB	12 (15.6)
	Mastectomy + SLNB + ALND	2 (2.6)
	Mastectomy + TAD	2 (2.6)
	Mastectomy	1 (1.3)
Postoperative pembrolizumab		
	Yes	34 (45.3)
	No	41 (54.7)
Median number of postoperative pembrolizumab cycles (range)	4 (1–9)	
Postoperative capecitabine or PARP inhibitor of 75 patients		
	Capecitabine	28 (37.3)
	Olaparib	2 (2.7)
Radiotherapy of 77 breasts		
	Yes	68 (88.3)
	No	9 (11.7)
Radiotherapy target of 77 breasts		
	Breast + lymph nodes	27 (39.7)
	Breast	12 (17.6)
	Breast + booster	9 (13.2)
	Breast + booster + lymph nodes	8 (11.8)
	Chest wall (after mastectomy) + lymph nodes	8 (11.8)
	Chest wall (after mastectomy)	2 (2.9)
	Breast + booster + lower axillary lymph nodes	1 (1.5)
	Supraclavicular lymph node area	1 (1.5)

PARP: Poly(ADP-ribose) polymerase enzyme

The median number of preoperative pembrolizumab cycles was 5 (range 1–8). Neoadjuvant pembrolizumab was discontinued due to AEs in 22 patients (29.3%). Of these, two patients received postoperative pembrolizumab.

All patients underwent surgery after recovery from AEs. Breast conserving surgery and sentinel lymph node biopsy was the most common type of surgery and chosen for 28 patients (36.4%).

Thirty-four patients (45.3%) were treated with adjuvant pembrolizumab. The median number of postoperative pembrolizumab cycles was 4 (range 1–9). Pembrolizumab was discontinued during the adjuvant phase in 8 (23.5%) of these 34 patients.

In 28 patients (37.3%) with residual disease capecitabine was included in the adjuvant therapy. Sixty-eight (88.3%) of the patients received radiotherapy. Radiotherapy was most often given to the breast and axillary lymph nodes (39.7%) including one patient with also supraclavicular area irradiation (1.5%). Three patients received postoperative endocrine therapy due to ER-positive residual tumors. In two patients, postoperative olaparib was started due to BRCA 1 mutation. One patient with HER2-positive IHC 3+ residual tumor was treated with adjuvant trastuzumab.

### Response to NAT

Response to NAT is shown in Table 3. A total of 42 patients (56.0%) achieved pCR in the pembrolizumab cohort, while pCR was seen in 47 patients (46.1%) of the cohort treated without pembrolizumab.

**Table 3:** Response to neoadjuvant treatment.

Residual cancer burden classification	NAT with pembrolizumab *n* (%)	NAT without pembrolizumab *n* (%)
RCB-0	42 (56.0)	47 (46.1)
RCB-I	9 (12.0)	12 (11.8)
RCB-II	13 (17.3)	27 (26.5)
RCB-III	10 (13.3)	8 (7.8)
Unknown	1 (1.3)	8 (7.8)

NAT: neoadjuvant therapy; RCB: Residual cancer burden.

In the pembrolizumab cohort, rCR was seen in 39 tumors (50.6%) and partial response (rPR) in 25 tumors (32.5%). The disease remained stable (rSD) in nine tumors (11.7%), while three tumors (3.9%) progressed. Radiologic response was statistically associated with pCR (*p* < 0.001). In the pembrolizumab cohort, all three progressive tumors were grade 3 with MIB1 scores 90–95%. One of these tumors had low HER-2 positivity by IHC, while the other two tumors were negative for HER2. In one tumor, 10% of the cells were ER-positive, while the other two were ER-negative.

### Association of baseline tumor and patient factors or treatment-related factors with pCR in the pembrolizumab cohort

pCR rates according to the tumor and patient characteristics of the pembrolizumab cohort are reported in Table 4. Lymph node metastasis (*p* = 0.011) and multifocality (*p* < 0.001) at baseline were negatively associated with achieving pCR with pembrolizumab. The multivariable analysis of multifocality, nodal involvement, primary radiological tumor size, grade, ER staining, HER2 status, and MIB1 score did not alter the results. Additionally, three out of four patients (75%) with metaplastic carcinoma did not achieve pCR. In the subgroup of 14 patients (18%) with ER-low tumors, pCR was seen in 8 patients (57%). ER status was not associated with pCR (*p* = 1.000). Tumor size was not associated with achieving pCR (*p* = 0.437). Younger patients achieved pCR more frequently than older patients but without statistical significance (*p* = 0.076).

**Table 4:** pCR rate according to the tumor and patient characteristics of the pembrolizumab cohort.

Characteristics	*n*	pCR rate (%)	*P*-value
Multifocality			
Yes	21 (27.3)	4 (19.0)	Fisher
No	56 (72.7)	39 (69.6)	
			*p* < 0.001
Nodal involvement			
Positive	34 (44.2)	13 (38.2)	Fisher
Negative	43 (55.8)	30 (69.8)	
			*p* = 0.011
Primary radiological tumor size			
cT1	20 (26.9)	10 (50.0)	
cT2	47 (61.0)	28 (59.6)	
cT3	8 (10.4)	5 (62.5)	
cT4	2 (2.6)	0	
			*p* = 0.437
Histology			
Invasive ductal carcinoma	71 (92.2)	41 (57.7)	
Invasive metaplastic carcinoma	4 (5.2)	1 (25.0)	
Invasive apocrine carcinoma	1 (1.3)	0	
Invasive lobular and ductal carcinoma	1 (1.3)	1 (100.0)	
			*p* = 0.253
Grade			
2	12 (15.6)	6 (50.0)	Fisher
3	65 (84.4)	37 (56.9)	
			*p* = 0.756
Estrogen receptor staining			
0	63 (81.8)	35 (55.6)	Fisher
1–10%	14 (18.2)	8 (57.1)	
			*p* = 1.000
HER2 status			
Negative	39 (50.6)	19 (48.7)	
1+	21 (27.3)	14 (66.7)	
2+	17 (22.1)	10 (58.8)	
			*p* = 0.450
MIB1 score			
Median (range)	80 (20–95)		Mann-Whitney
			*p* = 0.473
Menopausal status			
Premenopausal	39 (52.0)	24 (61.5)	
Postmenopausal	33 (44.0)	17 (51.5)	
Unknown	3 (4.0)	1 (33.3)	
			*p* = 0.476
The number of neoadjuvant pembrolizumab cycles			
1	3 (3.9)	1 (33.3)	
2	7 (9.1)	5 (71.4)	
3	5 (6.5)	2 (40.0)	
4	7 (9.1)	2 (28.6)	
5	20 (26.0)	13 (65.0)	
6	32 (41.6)	19 (59.4)	
7	1	0	
8	2 (2.6)	1 (50.0)	
			*p* = 0.531

pCR: pathologic complete response; HER2: human epidermal growth factor receptor 2; MIB-1: proliferation marker.

No statistical association was seen between the number of neoadjuvant pembrolizumab cycles and achieving pCR (*p* = 0.531).

### Event-free survival according to pCR

**Figure 2:**
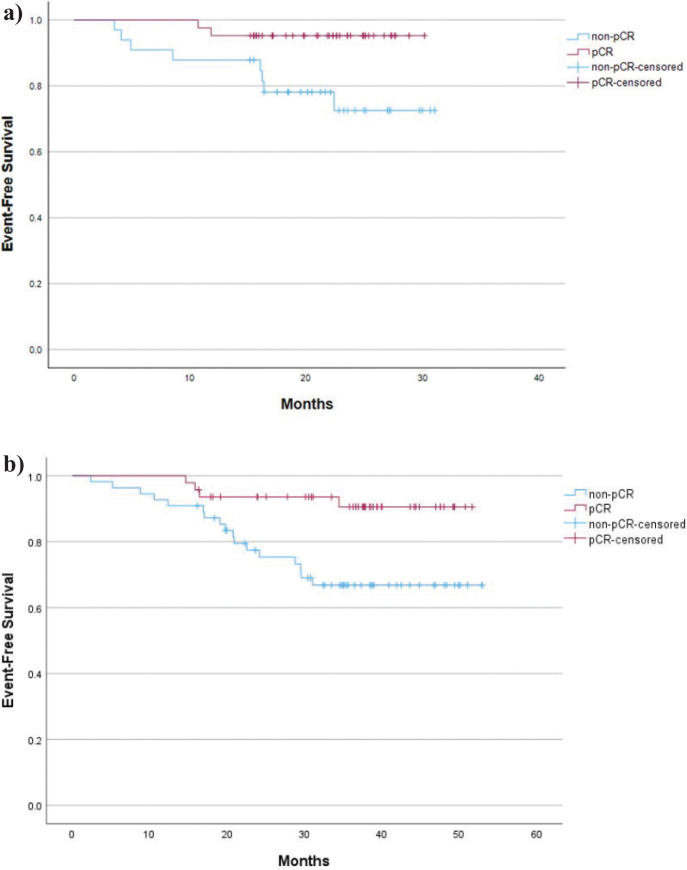
Event-free survival according to pCR of the patients treated with (a) neoadjuvant chemotherapy and pembrolizumab and (b) neoadjuvant chemotherapy without pembrolizumab. pCR: pathologic complete response.

In the pembrolizumab cohort, patients with pCR had longer EFS (*p* = 0.017) (Figure 2) than those with residual invasive cancer after surgery cohort. Median follow-up was 22 months (range 9–31). In the cohort with patients who did not receive pembrolizumab, patients with pCR had a longer EFS than those with residual invasive cancer after surgery (*p* = 0.006), and the median follow-up was 35 months (range 2–53).

### Treatment-related adverse events in the pembrolizumab cohort

Treatment-related toxicities of the pembrolizumab cohort are shown in Table 5. All patients had at least one AE of any grade. The most common grade 3 or 4 AE was decreased neutrophil count in 53 patients (70.7%). A total of 34 patients (45.3%) had irAEs. Hypothyroidism was the most common autoimmune disorder diagnosed in 14 patients (18.4%). Twenty-nine patients (38.7%) were hospitalized because of their AEs. The median length of hospital stay due to AEs was 5 days (range 1–13). None of the patients died due to AEs.

**Table 5:** Treatment-related adverse events of the patients treated with pembrolizumab.

Adverse event, *n* = 75 (all patients)	Any grade (% of all patients)	Grade 3 or 4 (% of all patients)
**Immune-related adverse event**	34 (45.3)	22 (29.3)
Hypothyroidism	14 (18.7)	0
Myocarditis	11 (14.7)	0
Hyperthyroidism	9 (12.0)	0
Hepatitis	6 (8.0)	6 (8.0)
Colitis	4 (5.3)	1 (1.3)
Nephritis	1 (1.3)	1 (1.3)
Adrenal insufficiency	3 (4.0)	1 (1.3)
Hypophysitis	1 (1.3)	1 (1.3)
**Other adverse events**		
Anemia	75 (100.0)	13 (17.3)
Neutrophil count decreased	73 (97.3)	53 (70.7)
Fatigue	69 (92.0)	0
Nausea	62 (82.7)	0
Elevated alanine aminotransferase level	51 (68.0)	9 (12.0)
Constipation	51 (68.0)	0
Diarrhea	43 (57.3)	4 (5.3)
Peripheral neuropathy	37 (49.3)	0
Rash	27 (36.0)	2 (2.7)
Myalgia	16 (21.4)	0
Headache	17 (22.7)	0
Vomiting	8 (10.7)	0
Febrile neutropenia	8 (10.7)	8 (10.7)
Arthritis	5 (6.7)	0
Arthralgia	3 (4.0)	0
**Hospitalization due to an adverse event**		
	Yes	29 (38.7)
	No	46 (61.3)
The median days spent in hospital (range)		5 (1–13)

Eleven patients (14.7%) had either a laboratory test or a radiological imaging indicating myocarditis. None of these were grade 3 or 4. The elevation of troponin appeared in average 159.4 ± 31.8 days after the initiation of the treatment with pembrolizumab, most often after the fifth treatment course. For the cardiac findings, see Table 6. Altogether 23 patients had a new troponin elevation under pembrolizumab treatment. In eight patients (34.8%), the troponin rise was observed prior to breast cancer surgery, while the elevation came postoperatively in 15 patients (65.2%). In all patients with troponin elevations, pembrolizumab treatment was discontinued permanently.

**Table 6:** Findings of patients with a new troponin elevation in the pembrolizumab cohort.

	All patients		Patients with ICI myocarditis		Patients without ICI myocarditis	
	*n* = 23		*n* = 11		*n* = 12	
	*n*	%	*n*	%	*n*	%
**Major criteria**						
CMR with definite myocarditis	5	21.7	5	45.5	0	0.0
**Minor criteria**						
CMR changes other than definite myocarditis	8	24.8	5	45.5	3	25.0
Extra cardiac ICI adverse events	8	24.8	5	45.5	3	25.0
Decline in cardiac function	2	8.7	2	18.2	0	0.0
Any new ECG change	7	30.4	4	36.4	3	25.0
Cardiac symptoms	4	17.4	3	27.3	1	8.3
Corticosteroid treatment	11	47.8	8	72.7	4	33.3

ICI: immune checkpoint inhibitor; ECG: electrocardiogram; CMR: cardiac magnetic resonance imaging.

The diagnostic criteria for ICI myocarditis were met in 11 patients (47.8%) with a new troponin rise. In 12 patients with troponin rise not meeting the ICI myocarditis criteria, anthracycline and capecitabine treatment were suspected to be the causative agent in three and one patients, respectively. Possible ICI myocarditis was suspected in three patients based on other autoimmune AE alone (one patient) or with nonspecific new ECG changes (two patients). In five patients, the troponin elevation remained unclear.

Six out of 11 patients (54.5%) with myocarditis achieved pCR. 16 out of 23 patients (70%) with diagnosed troponin rise and discontinued pembrolizumab achieved pCR.

### Discontinuation of pembrolizumab

Pembrolizumab was discontinued in 22 patients (29.3%) due to AEs in the preoperative setting, and in addition, 21 patients (28.0%) were not treated with adjuvant pembrolizumab because of earlier preoperative AEs, such as increased TnI levels. Additionally, adjuvant pembrolizumab was started but later discontinued due to AEs in eight of 34 patients (23.5%). Overall, in 51 patients (68.0%), pembrolizumab was discontinued due to AEs.

## Discussion and conclusion

pCR rate of 56% was seen in patients with early TNBC treated with NAT and pembrolizumab while in the cohort treated without pembrolizumab pCR rate was 46%.

Our RW pCR rate of 56% is in line with most other RW studies with pCR rate of 52–59% [19–23] but lower than the pCR rate of 64.8% in the landmark KEYNOTE-522 study [10] and also in a couple of RW studies with pCR rate of 63.6–64.7% [24, 25]. Unlike RW studies, the KEYNOTE-522 study was a randomized prospective phase 3 trial with strict inclusion and exclusion criteria. Therefore, the patient cohorts differ from each other, which may explain the higher pCR rate in the KEYNOTE-522 study.

Low ER-positive (1–9%) tumors were included in our and other RW studies but were not included in the landmark KEYNOTE-522 study. In our subgroup of 14 patients (18%) with ER-low tumors, the pCR rate of 57% was similar with the whole study population, which suggests the benefit of adding pembrolizumab. In fact, previous preclinical studies suggest that ER-low tumors are biologically more similar to ER-negative tumors, classified predominantly as basal-like or HER2 enriched by PAM50 intrinsic subtyping [28, 29]. In addition, ER-low tumors have also been shown to respond to NAT more similarly to ER-negative tumors [30, 31].

In the pembrolizumab cohort, of the investigated tumor biologic factors, multifocality and lymph node metastases were negatively associated with achieving pCR in both the univariate and multivariable analyses.

The size of the primary tumor had no impact on achieving pCR, which is in line with another RW study with 76 patients [32]. Notably, although our study included a high number of cT1 tumors, 30% of these were multifocal. In the KEYNOTE-522 study, T1–T2 tumors had a pCR rate of 70%, while pCR rate of 50% was seen in T3–T4 disease. Also, in a RW study with 153 patients, a higher probability for pCR was reported in stage I/II tumors compared to higher stages [25].

In our study, grade 3 or higher AEs were seen in 81% of the patients in our pembrolizumab cohort, which is in line with 82% of the patients in the KEYNOTE-522 study. Hospitalization rate of 38.7% in our pembrolizumab cohort is even higher than 26% seen in the large RW study from seven US academic medical center studies [19]. However, none of the patients died due to AEs.

Myocarditis was seen more often in our patients (14.7%) than in the KEYNOTE-522 study, where myocarditis occurred in three patients (0.4%) in the neoadjuvant setting and in two patients (0.4%) in the adjuvant setting. This discrepancy may partly be due to variations in different guidelines used for grading myocarditis. Higher myocarditis rates (5–7%) have also been seen in other RW studies [33, 34]. Notably, none of our patients had grade 3 or more serious cardiac AEs, and all these patients received a corticosteroid treatment course and recovered, but pembrolizumab was not given any more. Taken together, despite the higher incidence of myocarditis and interruption of the systemic therapy, higher pCR rates were seen both in our and the other similar RW study [33]. However, these findings underline the need for cardiac monitoring in patients receiving NAT including pembrolizumab.

Due to AEs, pembrolizumab was discontinued in the present study in 29% of the patients preoperatively, which is in line with other RW studies [19, 22, 23], while in the KEYNOTE-522 study, it was 16% [10]. We reported that additionally 28% of the patients did not start postoperative pembrolizumab and 11% of the patients discontinued postoperative pembrolizumab making the overall discontinuation rate as high as 68%. However, the number of preoperative pembrolizumab cycles was not associated with pCR in the present study. Moreover, the impact of adjuvant pembrolizumab after neoadjuvant pembrolizumab on survival outcomes has not been adequately addressed in clinical studies. In fact, in the supplementary Kaplan–Meier estimates of OS by pCR according to treatment group analyses of the KEYNOTE 522 study, the survival curves did not seem to differ [12]. This may indicate minor if any benefit for adjuvant pembrolizumab after pCR.

Backbone chemotherapy may also have influence on the effectiveness of NAT. A RW study with 153 early TNBC patients showed that dose reductions were associated with lower pCR rates. Specifically, patients receiving reduced doses of carboplatin had a pCR rate of 50% compared to those receiving full dose of carboplatin of 73% [24].

Predictive factors for pembrolizumab containing NAT for TNBC are needed to improve the outcome of this patient group and to save the patients with inadequate response from the AEs of ineffective therapies. Additionally, more clinical trials and real-world research combined with deep characterization of TNBC primary and residual tumors are needed to find tumor biology-related predictive factors and biological therapies for the patients without pCR and cure [35].

### Limitations

The present retrospective study was conducted at a single hospital and contains certain limitations. Firstly, our cohort is relatively small causing challenges in statistical analyses, especially in the subgroups. Secondly, the grading of AEs was often pending and had to be retrospectively evaluated according to the written notes by the clinical team. Thirdly, the follow-up period of the patients was short.

## Conclusions

In conclusion, patients who received NAT with pembrolizumab achieved pCR rate of 56%, while pCR rate of 46% was seen among those who did not receive pembrolizumab. To deliver pembrolizumab as planned is challenging due to their AEs. Even though the number of preoperative pembrolizumab cycles was not associated with pCR monitoring and limiting AEs is important. More studies are needed to better understand cardiovascular risks.

## Data Availability

Datasets generated and analyzed during the current study are not publicly available but are available from the corresponding author on reasonable request.
